# Dietary carotenoids and vitamin A are associated with lower gastric cancer risk and favorable inflammatory and oxidative stress biomarkers: an incident case– control study

**DOI:** 10.3389/fnut.2026.1953378

**Published:** 2026-09-07

**Authors:** Farhad Vahid, Torsten Bohn

**Affiliations:** Nutrition and Health Research Group, Department of Precision Health, Luxembourg Institute of Health, Strassen, Luxembourg

**Keywords:** absorption, apoptosis, carotenes, RAR, transcription factors, xanthophylls

## Abstract

**Background:**

Dietary carotenoids and vitamin A may contribute to gastric cancer (GC) prevention through anti-inflammatory and antioxidant mechanisms; however, epidemiological evidence remains limited. We investigated the associations of total and individual carotenoid and vitamin A intakes with GC and their relationships with inflammatory/oxidative stress biomarkers.

**Methods:**

The case–control study included 177 adults (82 GC incidence cases/95 controls). A validated 168-item food frequency questionnaire assessed dietary intakes of individual carotenoids and preformed vitamin A. Metabolic biomarkers, lipid profile, inflammatory/oxidative stress markers [e.g., high-sensitivity C-reactive protein (hs-CRP), tumor necrosis factor-alpha (TNF-*α*), interleukins (6/1*β*/10/4), total antioxidant capacity (TAC), and malondialdehyde (MDA)] were measured. Multivariable logistic regression models estimated odds ratios (ORs) for GC risk; linear regression models examined associations between carotenoid intakes and biomarkers.

**Results:**

Total carotenoid (mg/d; OR: 0.86, 95%CI: 0.81–0.98), *β*-carotene (mg/d; OR: 0.77, 95%CI: 0.65–0.90) and vitamin A (mg RAE/d; OR: 0.29, 95%CI: 0.09–0.95) intakes, as continuous variables, and also as binary variables (based on the median cut-offs), in all models (also adjusted for age, sex, smoking, education, marital status, physical activity, cancer history, *H. pylori*, NSAIDs intake, body mass index, total energy intake), were inversely associated with GC odds. In fully adjusted models, higher total carotenoid intake (mg/d) was associated with lower LDL-c (*β*: −1.60, 95%CI: −2.91, −0.25) and IL-6 (*β*: −7.92, 95%CI: −14.00, −2.00); lycopene (mg/d) with higher LDL-c (*β*: 2.030, 95%CI: 0.27, 4.31) and vitamin A intake (mg RAE/d) with lower TNF-*α* (*β*: −12.12, 95%CI: −24.71, −0.47) and higher TAC (*β*: 0.64, 95%CI: 0.001, 1.27).

**Conclusion:**

Higher intakes of total carotenoids and vitamin A were associated with lower odds of GC and more favorable profiles of inflammatory/oxidative stress biomarkers. These findings support an inverse association between carotenoid-rich dietary patterns and gastric carcinogenesis.

## 1 Introduction

Gastric cancer (GC) remains a major global health burden (1–3). In 2022, GC ranked among the top cancers worldwide, with roughly 969,000–1,089,000 new cases and approximately 660,000–769,000 deaths reported in recent global analyses, making it one of the leading causes of cancer mortality globally (1, 2). The disease accounts for a large proportion of cancer disability-adjusted life years (DALYs). Although age-standardized incidence and mortality rates have declined in many regions, the absolute numbers of cases and deaths remain high and are projected to increase with population growth and aging (1, 2). These burdens are unevenly distributed, with the highest incidence and mortality in Asia, parts of Latin America, and Eastern/Central Europe (1, 2).

GC is multifactorial, with established risk factors including *Helicobacter pylori* (*H. pylori*) infection [the single largest attributable cause worldwide; (4, 5)], tobacco use (2), high salt intake (6–8), low intake of fresh fruits and vegetables (6, 8), alcohol (5, 8), obesity (9), and certain occupational or environmental exposures, e.g., via air, water, and soil pollution, or radiation (10). Dietary patterns and specific nutrients, therefore, play plausible, potentially modifiable roles in gastric carcinogenesis, offering opportunities for primary prevention (8), in addition to constituting an accessible and affordable means for disease prevention across diverse populations.

Carotenoids include provitamin A compounds such as *β*-carotene, *α*-carotene, and *β*-cryptoxanthin, as well as others, such as lutein and lycopene. All of these have attracted attention for their antioxidant, anti-inflammatory, and immunomodulatory properties, which could interrupt key steps in gastric carcinogenesis driven by oxidative stress and chronic inflammation (11–13). *In vitro*, *in vivo*, and mechanistic studies have shown that carotenoids can scavenge reactive oxygen species [ROS, (14)], modulate NF-κB [nuclear factor kappa-light-chain-enhancer of activated B cells, (15)], and may enhance the body’s own antioxidant system by upregulating Nrf-2 (16), thus impacting inflammatory pathways related also to apoptosis and cell-cycle control (17). Provitamin A carotenoids and vitamin A may also affect cell proliferation via the molecular hormonal receptor RAR, which has been linked to cellular proliferation and apoptosis, with stronger activity favoring proliferation over apoptosis (18). Finally, carotenoids may mitigate the negative effects of *H. pylori* and improve digestive and gut flora (19).

Epidemiological findings are more mixed, but several case–control analyses and pooled reviews reported inverse associations with GC, particularly for *β*-carotene and *α*-carotene (12, 20–22), while cohort data and trials provide more heterogeneous results, and high-dose supplements have even shown harm in other cancer contexts, in part due to very high doses in conjunction with high bioavailability from some formulations (23–28). However, due to the strong relation of carotenoids to inflammation and oxidative stress, biomarkers of inflammation [e.g., interleukin (IL)-6, tumor necrosis factor-alpha (TNF-α), high-sensitivity C-reactive protein (hs-CRP)] and oxidative stress [total antioxidant capacity (TAC), malondialdehyde (MDA)] are considered meaningful markers (29), which could offer a mechanistic window to test whether carotenoid intake associates with biologically meaningful changes that could mediate GC risk.

Despite biological plausibility and some supportive observational data, important gaps remain. Many prior studies have been limited by poor dietary assessment, heterogeneity in exposure definitions (dietary intake versus blood levels), inadequate control for *H. pylori* and other confounders, and limited integration of dietary data with inflammatory and oxidative biomarkers to clarify mechanistic aspects (25, 28). In addition, evidence distinguishing effects of specific carotenoids (rather than total carotenoids) on GC risk and on proximate biomarker signatures is sparse, especially in populations with differing *H. pylori* prevalence and dietary patterns (28, 30). These gaps limit causal inference and the translation of findings into prevention guidance.

We hypothesized that higher dietary intakes of total and individual carotenoids and vitamin A are associated with lower odds of GC and a more favorable profile of inflammatory and oxidative stress biomarkers. Unlike previous studies that primarily focused on overall carotenoid exposure or cancer outcomes alone, our approach was designed to identify carotenoid-specific associations, providing mechanistic insights into whether modulating inflammation and oxidative stress as assessed by biomarker profiling may underlie the relationship between carotenoid intake and gastric carcinogenesis, bridging the gap between epidemiological association and mechanistic aspects of carotenoids. The present case–control study aimed thus to (a) examine associations between dietary intakes of individual and total carotenoids and vitamin A and odds of GC, and (b) evaluate associations between these intakes and circulating metabolic, inflammatory, and oxidative stress biomarkers to assess mechanistic signatures linking individual and total carotenoid exposure to GC, potentially contributing to evidence that will inform nutritional strategies for GC prevention.

## 2 Materials and methods

### 2.1 Study design and participants

This hospital-based case–control investigation was conducted at specialized healthcare centers in Northwest Iran between December 2014 and May 2016. The study population included 82 cases diagnosed with GC and 95 control participants. Cases were newly diagnosed GC patients (diagnosed within the preceding month) identified by a gastroenterologist and referred to the research team. Participants were selected through simple random sampling: an exhaustive list of eligible patients was prepared, and each individual had an equal probability of being selected. Control participants were randomly recruited from caregivers of patients attending the same clinics and were frequency-matched to cases by age (±5 years) and sex (1:1). Data collection for both cases and controls occurred concurrently in the same clinical settings. All participants provided written informed consent following verbal and written explanation of the study procedures. The study protocol received ethical approval from the Shahid Beheshti University of Medical Sciences Ethics Review Committee, Tehran, Iran, in accordance with the 2008 Declaration of Helsinki (approval date: 8 December 2012; tracking number: 9773085; project code: 647). The study, design, and methods are published in detail elsewhere (31).

### 2.2 Eligibility criteria

Inclusion criteria comprised: (a) absence of any malignancy (for control participants), (b) no adherence to specialized diets, including vegetarian or weight-modifying diets, within the year prior to enrollment, (c) no history of pregnancy, lactation, cancer (for controls), or major neurological, gastrointestinal, hepatic, endocrine, immune, renal, or cardiovascular diseases, (d) age between 20 and 80 years, and (e) willingness to participate in the study. Participants were excluded if they failed to comply with study procedures, made significant dietary changes during the study period (e.g., weight-altering diets), or reported implausible energy intakes (<800 or >5,500 kcal/day, equivalent to ±3SD deviations from study population mean).

### 2.3 Blood collection and assessment of inflammatory markers

#### 2.3.1 Participant preparation

Participants were instructed to avoid corticosteroids, anti-inflammatory medications, and analgesics for at least 48 h prior to blood collection.

### 2.4 Blood sampling

After an overnight fast of 10–12 h, 10 mL of venous blood was drawn under sterile conditions between 08:30 and 10:30 a.m. Serum was separated immediately by centrifugation and stored at −70 °C until analysis. Serum concentrations of inflammatory markers, including TNF-*α*, IL-1*β*, IL-4, IL-6, IL-10, and hsCRP, were quantified using ELISA kits from Pishtaz Teb Zaman Diagnostics Co., Ltd. (Tehran, Iran) and Shanghai Crystal Day Biotech Co., Ltd., supplied by Negin Salamat Saba Co. *H. pylori* infection status was determined via *H. pylori*-specific IgG measurement using a commercial ELISA kit (Shanghai Crystal Day Biotech Co., Ltd., Shanghai, China).

### 2.5 Dietary assessment

Dietary intake over the past year was evaluated using a semi-quantitative food frequency questionnaire (FFQ) that assessed 168 food items (32). Participants reported consumption frequency using standardized portion sizes. Case participants were instructed to reflect on their diet prior to GC diagnosis. Intake frequencies were recorded as daily, weekly, monthly, yearly, or never, depending on the food item. Nutrient and energy intake, including carotenoids and preformed vitamin A, was computed using Nutritionist IV software (First Databank, Hearst Corp., San Bruno, CA, USA). To calculate nutrient intakes based on reported food consumption, the USDA food database (33) was used.

### 2.6 Assessment of other variables

Information on demographics, lifestyle, and clinical factors, including age, sex, smoking status, alcohol consumption, NSAID use, physical activity, education level, and family history of cancer, was collected through structured interviews. Anthropometric measurements were performed by trained personnel: weight was measured with light clothing using a SECA digital scale to the nearest 0.1 kg, and height was recorded without shoes against a wall-mounted tape measure to the nearest 0.5 cm. Body mass index (BMI) was calculated as weight (kg) divided by height squared (m^2^).

## 3 Statistical analysis

Descriptive statistics were calculated using independent *t*-tests for continuous variables and chi-square tests for categorical variables. Associations between carotenoids and vitamin A (as dichotomous variables) and participant characteristics, including age, sex, BMI, education, smoking, *H. pylori* infection, physical activity, aspirin/NSAID use, and family history of cancer, were examined. Linear regression models estimated beta-coefficients and 95% confidence intervals (CIs) for associations with inflammatory and oxidative stress biomarkers, while logistic regression models estimated odds ratios (ORs) and 95% CIs for GC risk. Both carotenoids and vitamin A were analyzed as continuous variables and dichotomized at the median (the sample-specific median cut-offs are analytical rather than clinical thresholds). Models were run unadjusted, adjusted for age and sex, and further adjusted for BMI, education, smoking, *H. pylori* infection, physical activity, aspirin/NSAID use, and total energy intake. The individual carotenoid variables were not entered simultaneously into a single regression model; rather, each carotenoid was evaluated separately, as strong correlations were expected among certain carotenoids. Therefore, potential multicollinearity among the carotenoid variables did not affect the estimated associations within the individual models. Statistical analyses were conducted using SPSS version 25 (IBM Corp., Armonk, NY, USA), with a *p*-value of 0.05 indicating statistical significance (two-sided). To control the false discovery rate resulting from multiple testing, multiple-comparison adjustments were performed using the Benjamini-Hochberg procedure. All reported *p*-values presented in the main and supplementary analyses are Benjamini-Hochberg adjusted. There were no missing data for the variables included in the final regression models; therefore, no imputation was performed, and analyses were conducted using complete observations.

## 4 Results

### 4.1 Baseline characteristics of the study population

Baseline demographic, clinical, and biochemical characteristics of GC cases and controls, as well as participants stratified by median (sample-specific cut-offs) intakes of total carotenoids and vitamin A, are presented in Table 1. Cases and controls were comparable with respect to age, sex distribution, smoking status, education level, marital status, regular aspirin or NSAID use, and immediate family history of cancer. However, cases had significantly higher BMI and fasting blood sugar (FBS) levels compared to controls. Cases also exhibited significantly higher low-density lipoprotein-cholesterol (LDL-c) concentrations, while no significant differences were observed for high-density lipoprotein-cholesterol (HDL-c), total cholesterol, or triglycerides (Table 1).

**Table 1:** Distribution [mean ± SD or *n* (%)] of participants’ characteristics across case–control, total carotenoids, and vitamin A intake groups.

Characteristics	Cases (*n* = 82)	Controls (*n* = 95)	*p*-values*	Total carotenoids (mg/d) (*n* = 89) ≤ 14.1**	Total carotenoids (mg/d) (*n* = 88) > 14.1**	*p*-values*	Vitamin A (mg RAE/day) (*n* = 90) ≤ 0.58**	Vitamin A (mg RAE/day) (*n* = 87) > 0.58**	*p*-values*	Total
Age (year)	51.4 ± 11.8	48.3 ± 10.5	0.076	51.0 ± 11.1	48.5 ± 11.5	0.144	50.9 ± 11.7	48.6 ± 10.8	0.170	49.7 ± 11.3
BMI (kg/m^2^)	26.4 ± 5.1	25.0 ± 2.7	**0.021**	26.1 ± 4.7	25.1 ± 3.3	0.096	26.1 ± 4.4	25.2 ± 3.7	0.140	25.6 ± 4.1
FBG (mg/dL)	97.5 ± 36.3	89.5 ± 13.3	**0.046**	94.1 ± 32.0	92.3 ± 20.4	0.648	93.3 ± 24.4	93.1 ± 29.1	0.968	93.2 ± 26.7
HDL-c (mg/dL)	46.5 ± 9.9	47.5 ± 12.2	0.552	46.2 ± 10.7	47.9 ± 11.6	0.333	46.2 ± 11.7	47.9 ± 10.6	0.310	47.1 ± 11.2
LDL-c (mg/dL)	109 ± 33.8	94.9 ± 25.8	**0.002**	106 ± 30.7	97.3 ± 30.0	0.060	104 ± 31.1	98.73 ± 29.9	0.222	102 ± 30.6
Cholesterol (mg/dL)	181 ± 39.8	174 ± 33.3	0.209	183 ± 36.7	172 ± 35.8	0.053	178 ± 32.2	177 ± 38.1	0.995	178 ± 36.5
TG (mg/dL)	140 ± 57.0	136 ± 58.6	0.636	139 ± 54.4	138 ± 61.2	0.947	145 ± 56.3	132 ± 58.7	0.141	138 ± 57.7
hsCRP (mg/L)	2.39 ± 1.1	1.82 ± 1.0	**0.001**	2.12 ± 1.2	2.05 ± 1.0	0.625	2.14 ± 1.1	2.04 ± 1.1	0.576	2.09 ± 1.1
IL-6 (pg/mL)	235 ± 172	103 ± 61.4	**<0.001**	188 ± 161	139 ± 115	**0.019**	172 ± 139	155 ± 144	0.419	164 ± 142
IL-1*β* (pg/mL)	2.8 ± 1.5	1.8 ± 0.9	**<0.001**	2.4 ± 1.5	2.1 ± 1.2	0.110	2.3 ± 1.4	2.2 ± 1.3	0.659	2.3 ± 1.3
TNF-*α* (pg/mL)	42.0 ± 29.1	19.1 ± 16.1	**<0.001**	30.8 ± 25.8	28.6 ± 25.7	0.569	33.2 ± 29.9	23.1 ± 20.1	0.066	29.7 ± 25.7
IL-10 (pg/mL)	1.9 ± 1.4	3.0 ± 2.0	**<0.001**	2.5 ± 1.7	2.5 ± 2.0	0.802	2.5 ± 1.9	2.5 ± 1.8	0.980	2.5 ± 1.8
IL-4 (pg/mL)	11.7 ± 12.5	9.0 ± 7.2	0.105	8.9 ± 10.0	11.3 ± 9.9	0.111	10.0 ± 10.5	10.3 ± 9.6	0.800	10.2 ± 10.0
TAC (μM)	1.2 ± 1.3	1.9 ± 1.3	**<0.001**	1.5 ± 1.2	1.7 ± 1.4	0.378	1.4 ± 1.3	1.7 ± 1.3	0.214	1.6 ± 1.3
MDA (μM)	3.7 ± 2.2	3.0 ± 1.7	**0.017**	3.4 ± 1.9	3.3 ± 2.1	0.816	3.4 ± 2.1	3.3 ± 1.9	0.761	3.3 ± 2.0
Sex (women)	45 (54.8%)	52 (54.7%)	0.985	48 (53.9%)	49 (55.6%)	0.763	55 (61.1%)	42 (48.3%)	0.071	97 (54.8%)
Smoking (Yes)	14 (17.1%)	15 (15.8%)	0.841	15 (16.8%)	14 (15.9%)	0.999	16 (17.7%)	13 (14.9%)	0.687	29 (16.4%)
High school diploma or lower	51 (62.2%)	67 (70.5%)	0.226	61 (68.5%)	57 (64.7%)	0.634	62 (68.8%)	56 (64.3%)	0.633	118 (66.7%)
Single	16 (19.5%)	14 (14.7%)	0.644	13 (14.6%)	17 (19.3%)	0.751	11 (12.2%)	19 (21.8%)	0.133	30 (16.9%)
Married	61 (74.3%)	73 (76.8%)		69 (77.5%)	65 (73.8%)		74 (82.2%)	60 (68.9%)		134 (75.7%)
Other (divorced, widowed, …)	5 (6.1%)	8 (8.4%)		7 (7.8%)	6 (6.8%)		5 (5.5%)	8 (9.2%)		13 (7.3%)
Regular physical activity (Yes)	14 (17.1%)	30 (31.6%)	**0.036**	22 (24.7%)	22 (25.0%)	0.999	22 (24.4%)	22 (25.3%)	0.999	44 (24.9%)
Cancer history (yes)***	13 (15.8%)	11 (11.6%)	0.510	15 (16.8%)	9 (10.2%)	0.272	11 (12.2%)	13 (14.9%)	0.664	24 (13.6%)
*H. pylori* (+)	61 (74.4%)	49 (51.6%)	**0.002**	68 (70.4%)	42 (47.7%)	**<0.001**	61 (67.7%)	49 (56.3%)	0.124	110 (62.1%)
Aspirin or NSAIDs intake (yes)	7 (8.5%)	9 (9.5%)	0.999	10 (11.2%)	6 (6.8%)	0.433	11 (12.2%)	5 (5.7%)	0.190	16 (9.0%)

* All *p*-values were adjusted using the Benjamini–Hochberg correction method. *p*-values reaching statistical significance are shown in bold type.

** Median (sample-specific cut-offs).

*** Immediate family members’ cancer history.

BMI: body mass index; FBG: fasting blood sugar; HDL-c: high-density lipoprotein cholesterol; LDL-c: low-density lipoprotein cholesterol; TG: triglycerides; hsCRP: high-sensitivity C-reactive protein; IL: interleukin; TNF-α: tumor necrosis factor alpha; TAC: total antioxidant capacity; MDA: malondialdehyde.

Regarding inflammatory and oxidative stress biomarkers, GC cases showed significantly higher levels of hs-CRP, IL-6, IL-1*β*, TNF-*α*, and MDA, and significantly lower levels of IL-10 and TAC than controls. No significant difference was observed for IL-4 between groups. In addition, the prevalence of *H. pylori* infection was significantly higher among cases than controls, whereas controls reported significantly higher engagement in regular physical activity (Table 1).

When participants were stratified by median (sample-specific cut-offs) intake of total carotenoids (≤14.1 mg/d *vs.* >14.1 mg/d), individuals with higher intakes were less likely to be *H. pylori*–positive and had significantly lower IL-6 concentrations compared to those below the median. Other demographic, metabolic, inflammatory, and oxidative stress variables did not differ significantly between total carotenoid intake groups. Similarly, when stratified by median preformed vitamin A intake (≤0.58 mg/d vs. >0.58 mg/d, as retinoic acid equivalents or RAE), no significant differences were observed in demographic characteristics, lifestyle factors, lipid profile, glycemic markers, inflammatory cytokines, oxidative stress markers, or *H. pylori* status between intake groups (Table 1).

### 4.2 Dietary intakes of participants

**Figure 1:**
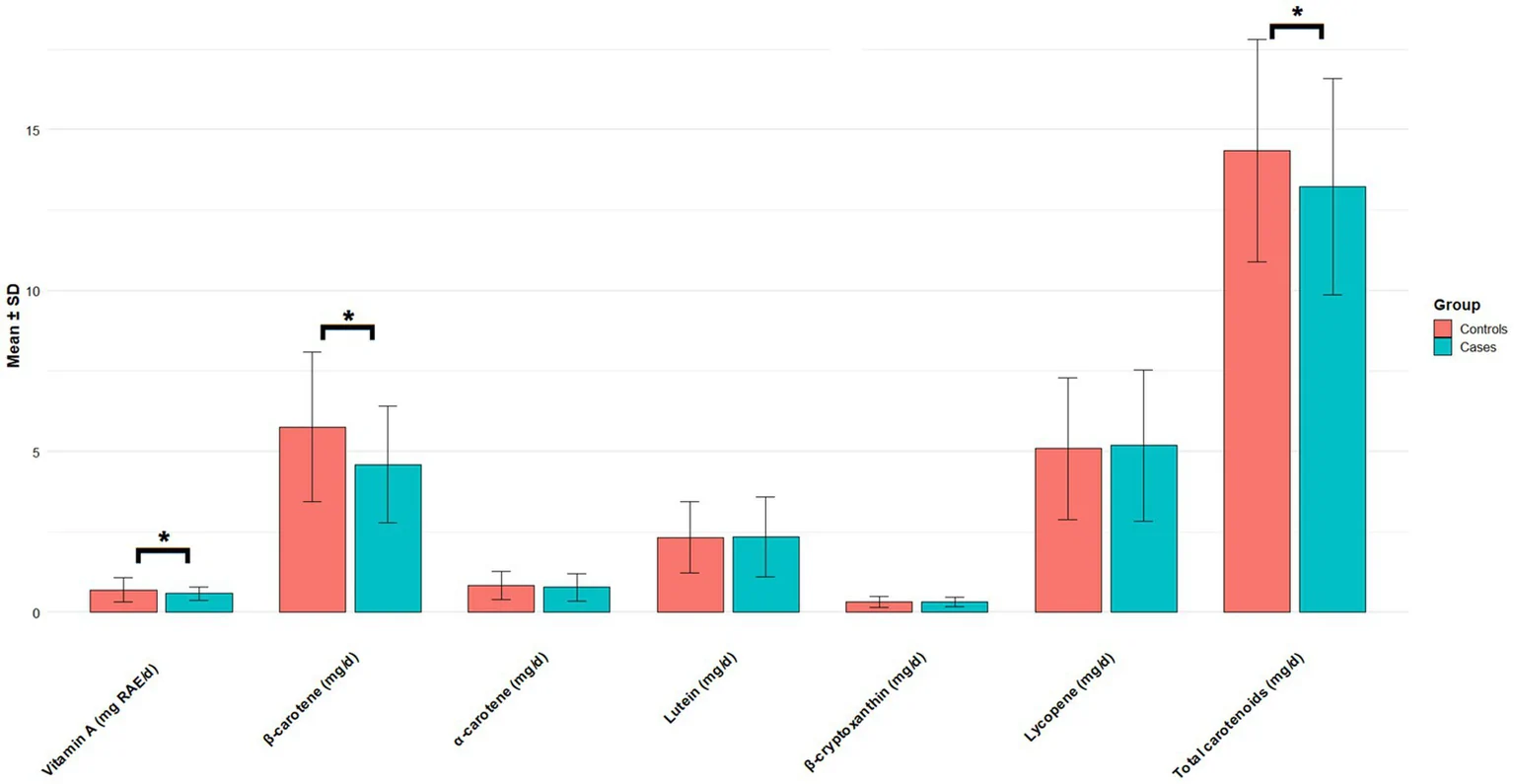
Mean dietary carotenoid intakes in controls and patients with gastric cancer. Bars show mean intake ± SD of 82 cases and 95 controls. * Significant difference between cases and controls.

Total energy intake did not differ significantly between cases and controls (Table 2). However, cases reported significantly higher total fat intake compared to controls. Compared with controls, GC cases had significantly lower intakes of vitamin A, *β*-carotene, and total carotenoids (Figure 1). This is also reflected in the lower intake of fruits among GC cases. Intakes of *α*-carotene, lutein, *β*-cryptoxanthin, lycopene, vegetables, legumes, starchy vegetables, and fish were similar between groups (Table 2).

**Table 2:** Distribution [mean ± SD or *n* (%)] of participants’ dietary intakes across case–control, total carotenoids, and vitamin A intake groups.

Characteristics	Cases (n = 82)	Controls (n = 95)	*p*-values*	Total carotenoids (mg/d) (*n* = 89) ≤ 14.1 **	Total carotenoids (mg/d) (*n* = 88) > 14.1**	*p*-values*	Vitamin A (mg RAE/day) (*n* = 90) ≤ 0.58**	Vitamin A (mg RAE/day) (*n* = 87) > 0.58**	*p*-values*	Total
Energy (kcal/d)	3,012 ± 625	2,991 ± 549	0.806	3,116 ± 612	2,887 ± 533	**0.009**	3,021 ± 597	2,981 ± 573	0.653	3,001 ± 584
Total fat (g/d)	120 ± 42.9	107 ± 32.6	**0.022**	120 ± 44.9	105 ± 28.5	**0.009**	116 ± 42.2	109 ± 33.4	0.248	112 ± 38.2
Vitamin A (mg RAE/day)	0.59 ± 0.20	0.70 ± 0.38	**0.018**	0.64 ± 0.28	0.65 ± 0.34	0.862	0.43 ± 0.10	0.87 ± 0.30	**<0.001**	0.65 ± 0.31
*β*-Carotene (mg/day)	4.59 ± 1.82	5.76 ± 2.32	**<0.001**	4.15 ± 1.65	6.30 ± 2.12	**<0.001**	5.05 ± 2.11	5.38 ± 2.23	0.318	5.22 ± 2.18
α-Carotene (mg/day)	0.78 ± 0.42	0.83 ± 0.44	0.472	0.71 ± 0.35	0.91 ± 0.48	**0.002**	0.78 ± 0.42	0.83 ± 0.44	0.471	0.81 ± 0.43
Lutein (mg/day)	2.35 ± 1.25	2.34 ± 1.11	0.929	2.04 ± 0.80	2.65 ± 1.40	**<0.001**	2.29 ± 1.05	2.39 ± 1.28	0.553	2.34 ± 1.17
*β*-Cryptoxanthin (mg/day)	0.33 ± 0.15	0.34 ± 0.17	0.622	0.32 ± 0.15	0.35 ± 0.17	0.259	0.32 ± 0.17	0.35 ± 0.15	0.236	0.33 ± 0.16
Lycopene (mg/day)	5.20 ± 2.35	5.10 ± 2.20	0.779	3.93 ± 1.75	6.36 ± 2.10	**<0.001**	5.09 ± 2.34	5.18 ± 2.20	0.787	5.14 ± 2.27
Total carotenoids (mg/day)	13.23 ± 3.36	14.36 ± 3.46	**0.031**	11.15 ± 2.12	16.56 ± 2.15	**<0.001**	13.76 ± 3.67	13.91 ± 3.24	0.777	13.84 ± 3.45
Legumes (g/day)	50.1 ± 29.1	50.4 ± 26.1	0.949	47.7 ± 25.3	52.8 ± 29.3	0.215	48.6 ± 25.8	52.0 ± 28.9	0.412	50.3 ± 27.4
Starchy vegetables (g/day)	19.7 ± 16.8	19.4 ± 16.4	0.911	17.2 ± 15.0	21.9 ± 17.7	0.057	19.0 ± 14.9	20.0 ± 18.1	0.705	19.5 ± 16.5
Fish (g/day)	7.0 ± 8.5	7.1 ± 8.3	0.930	6.4 ± 6.5	7.6 ± 10.0	0.373	5.6 ± 5.7	8.5 ± 10.3	**0.022**	7.0 ± 8.4
Lipids (g/day)	32.9 ± 15.4	25.5 ± 16.1	**0.002**	30.0 ± 17.4	27.9 ± 14.8	0.422	28.2 ± 14.5	29.6 ± 17.8	0.575	28.9 ± 16.2
Vegetables (g/day)	404 ± 147	436 ± 139	0.145	419 ± 140	423 ± 147	0.842	406 ± 138	437 ± 148	0.157	422 ± 143
Fruits (g/day)	373 ± 115	419 ± 108	**0.007**	397 ± 105	398 ± 122	0.971	397 ± 101	398 ± 101	0.944	398 ± 114

* All *p*-values were adjusted using the Benjamini–Hochberg correction method. *p*-values reaching statistical significance are shown in bold type.

** Median (sample-specific cut-offs).

When participants were stratified by median (sample-specific cut-offs) total carotenoid intake, those with higher intakes had significantly lower energy and total fat intake and higher intake of *β*-carotene, α-carotene, lutein, and lycopene than those below the median. No significant differences were observed for vitamin A, *β*-cryptoxanthin, or intake of legumes, vegetables, fruits, fish, or starchy vegetables (Table 2).

Stratification by median vitamin A intake showed that participants with higher vitamin A intake did not differ significantly from those below the median in total energy intake, macronutrient intake, individual carotenoids, total carotenoids, or food group consumption, with the exception of fish intake, which was significantly higher among those with vitamin A intake above the median (Table 2).

### 4.3 Associations between carotenoid and vitamin A intake and odds of GC

**Figure 2:**
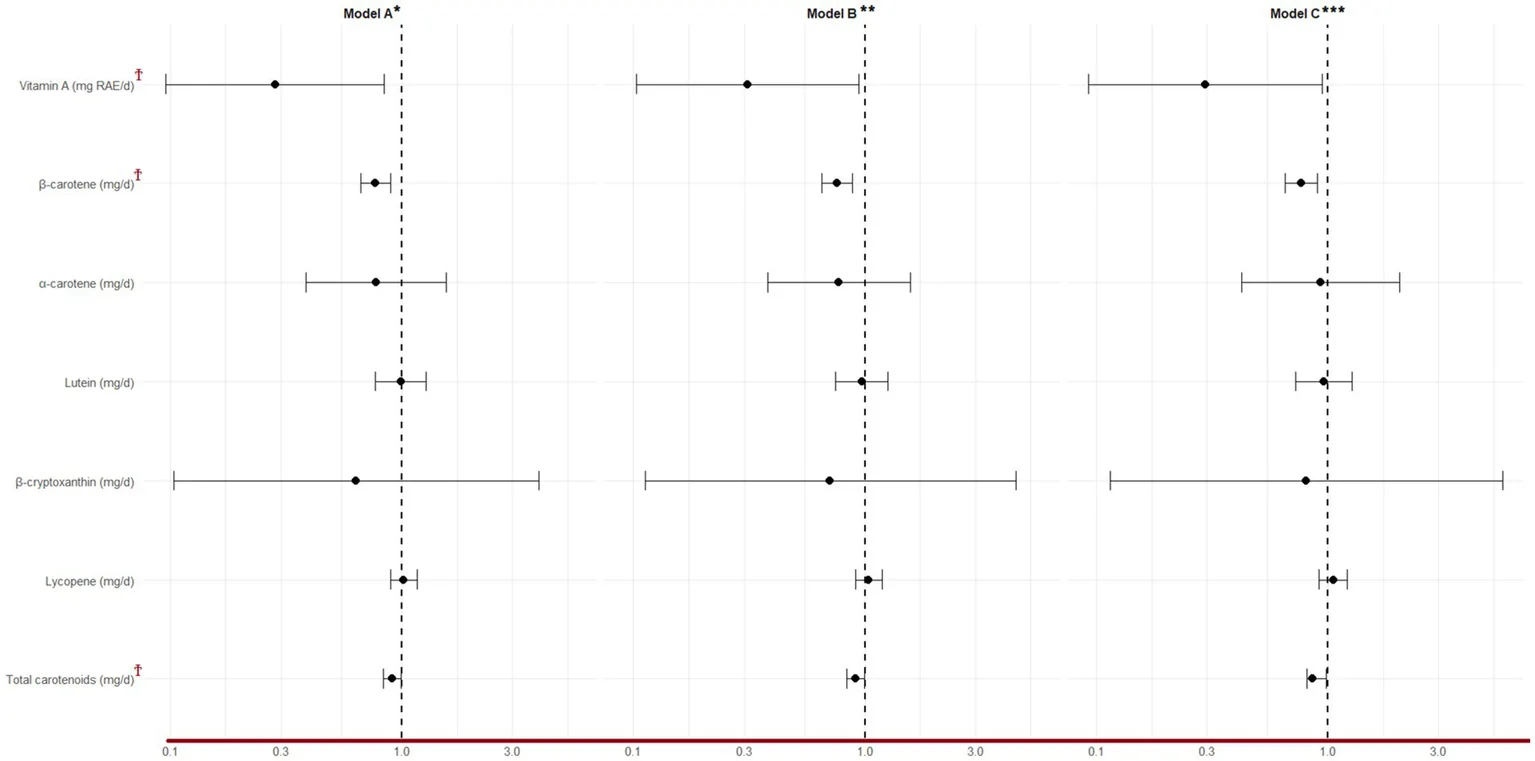
Association between carotenoid (mg/d) and vitamin A (mg RAE/d) intakes as continuous variables and odds (95% CI) of gastric cancer based on three logistic regression models. *Crude model, without any further adjustment. ** Adjusted for age and sex. *** Adjusted for age, sex, smoking (yes/no), education (lower than high school diploma/higher than higher school diploma), marital status (married/single/others), having regular physical activity (yes/no), immediate family members’ cancer history (yes/no), *H. pylori* (+/−), regular aspirin or NSAIDs intake (yes/no), BMI, and total energy intake (kcal/day). *^Ϯ^* Significant in all three models after Benjamini-Hochberg correction.

In crude multivariable logistic regression models, higher intakes of total carotenoids (OR = 0.91, *p* = 0.033), *β*-carotene (OR = 0.77, *p* < 0.001), and vitamin A (OR = 0.28, *p* = 0.023), analyzed as continuous variables, were significantly associated with lower odds of GC (Figure 2 and Supplementary Table 1). These inverse associations remained statistically significant after adjustment for age and sex (Model B), and additional adjustment for lifestyle and socioeconomic factors, including smoking status, education, marital status, physical activity, family history of cancer, *H. pylori* infection, aspirin or NSAID use, BMI, and total energy intake (Model C: fully adjusted model). Specifically, in fully adjusted models, total carotenoid intake (OR: 0.86, *p* = 0.034), *β*-carotene intake (OR: 0.77, *p* < 0.001), and vitamin A intake (OR: 0.29, *p* = 0.040) were inversely associated with GC risk. No statistically significant associations were observed for other carotenoids across any of the models when analyzed as continuous variables (Figure 2 and Supplementary Table 1).

**Figure 3:**
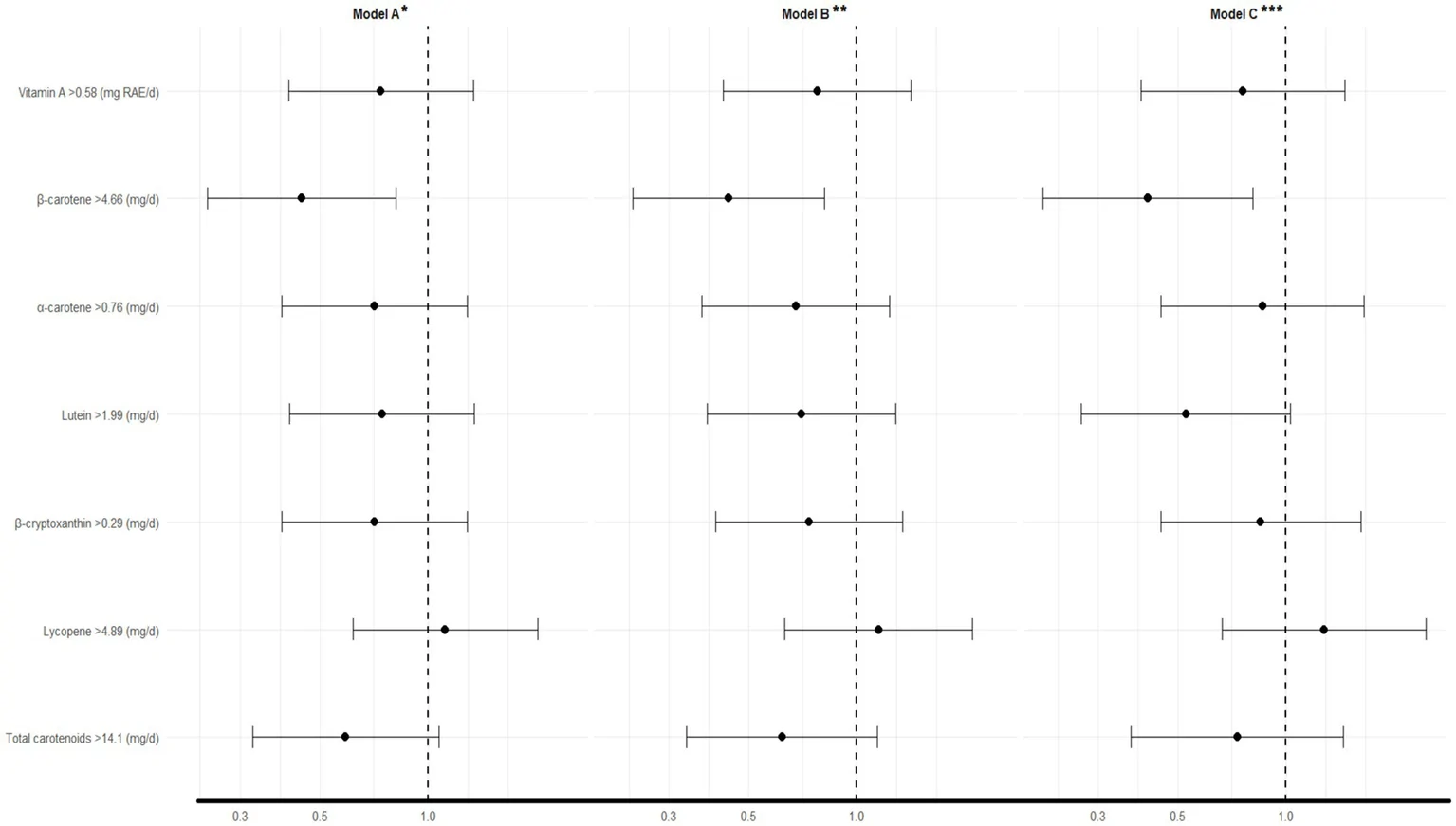
Odds ratios (95% CI) for gastric cancer comparing high versus low (median-based) carotenoid and vitamin A intake across three logistic regression models. * Crude model, without any further adjustment. ** Adjusted for age and sex. *** Adjusted for age, sex, smoking (yes/no), education (lower than high school diploma/higher than higher school diploma), marital status (married/single/others), having regular physical activity (yes/no), immediate family members’ cancer history (yes/no), *H. pylori* (+/−), regular aspirin or NSAIDs intake (yes/no), BMI, and total energy intake (kcal/day).

When dietary intakes were analyzed as categorical variables based on median (sample-specific cut-offs), only participants with intakes above the median of *β*-carotene had significantly lower odds of GC compared to those below the median in crude (OR: 0.45, *p* = 0.009), age and sex adjusted (OR: 0.44, *p* = 0.009) and fully adjusted models (OR: 0.41, *p* = 0.010, Figure 3, and Supplementary Table 1).

### 4.4 Association between carotenoid and vitamin A intake and biomarkers

#### 4.4.1 Crude models

In crude models (without any adjustments), based on linear regression models, higher intakes of total carotenoids (continuous variables) were significantly associated with HDL-c (*β*: 1.40, *p* = 0.050) and IL-6 (*β*: −8.30, *p* = 0.007). In addition, there was a significant association between higher intakes of *β*-carotene and lower TNF-*α* (*β*: −1.75, *p* = 0.049) and higher intakes of *β*-cryptoxanthin and lower HDL-c (*β*: −10.40, *p* = 0.043) and higher IL-4 (*β*: 9.25, *p* = 0.045), and higher intake of lycopene with higher LDL-c (*β*: 2.02, *p* = 0.047); and also, higher intake of lutein and higher total cholesterol (*β*: 5.06, *p* = 0.031, Supplementary Table 2).

In addition, in crude linear regression models, there was a significant inverse association between higher-than-median intake of total carotenoids and *β*-carotene and lower IL-6 (*β*: −49.6, *β*: −44.8, *p* = 0.019, *p* = 0.035). There was also a positive, significant association between higher-than-median lycopene intake and higher LDL-c (*β*: 11.1, *p* = 0.015, Supplementary Table 3).

#### 4.4.2 Adjusted for age and sex

After controlling for age and sex, there was a significant association (linear regression models) between total carotenoids and lower LDL-c (*β*: −1.54, *p* = 0.020) and lower IL-6 (*β*: −7.50, *p* = 0.014) and between *β*-carotene and lower TNF-*α* (*β*: −1.76, *p* = 0.049) and *β*-cryptoxanthin and lower HDL-c (*β*: −11.24, *p* = 0.029). On the other hand, there was a significant positive association between vitamin A and TAC (*β*: 0.63, *p* = 0.048), between *β*-cryptoxanthin and IL-4 (*β*: 9.76, *p* = 0.036), and between lycopene and LDL-c (*β*: 2.41, *p* = 0.017; Supplementary Table 4).

In age- and sex adjusted linear regression models, there was a significant association between higher-than-median intake of total carotenoids and lower LDL-c (*β*: −10.9, *p* = 0.017) and IL-6 (*β*: −42.3, *p* = 0.047), and between *β*-carotene and lower IL-6 (*β*: −43.1, *p* = 0.040). On the other hand, there was a positive and significant association between higher-than-median lycopene (>4.89 mg/d) and LDL-c (*β*: 11.3, *p* = 0.012, Supplementary Table 5).

#### 4.4.3 Fully adjusted models

For the fully adjusted models (adjusted for age, sex, smoking, education, marital status, having regular physical activity, immediate family members’ cancer history, *H. pylori*, regular aspirin or NSAIDs intake, BMI, and total energy intake), when carotenoids and vitamin A were entered in the models as continuous variables, there was an inverse and significant association between total carotenoid intakes and LDL-c (*β*: −1.60, *p* = 0.020) and IL-6 (*β*: −7.92, *p* = 0.010) and between vitamin A with TNF-α (*β*: −12.12, *p* = 0.049) and *β*-cryptoxanthin with HDL-c (*β*: −10.38, *p* = 0.030). On the other hand, there was a positive, significant association between vitamin A and TAC (*β*: 0.64, *p* = 0.044), between *β*-cryptoxanthin and IL-4 (*β*: 8.61, *p* = 0.032), and between lycopene and LDL-c (*β*: 2.30, *p* = 0.020; Table 3).

**Table 3:** Association (*β*-coefficients, 95%CI) between carotenoid and vitamin A intakes and metabolic, inflammatory, and oxidative stress biomarkers, as determined by linear regression models^§^.

Variables**	Vitamin A (mg RAE/day)	*p*-values*	*β*-carotene (mg/d)	*p*-values*	α-carotene (mg/d)	*p*-values*	Lutein (mg/d)	*p*-values*	*β*-cryptoxanthin (mg/d)	*p*-values*	Lycopene (mg/d)	*p*-values*	Total carotenoids (mg/d)	*p*-values*
FBG (mg/dL)	−0.58 (−13.74, 12.58)	0.855	−0.03 (−1.92, 1.86)	0.738	0.81 (−8.56, 10.37)	0.936	−0.94 (−4.64, 2.76)	0.339	−0.24 (−25.20, 24.71)	0.989	0.68 (−1.16, 2.52)	0.485	−0.20 (−1.41, 1.02)	0.948
HDL-c (mg/dL)	−0.01 (−5.48, 5.47)	0.909	−0.40 (−1.17, 0.39)	0.245	−0.67 (−4.64, 3.30)	0.617	1.00 (−0.54, 2.52)	0.323	−10.38 (−20.63, −0.14)	**0.030**	−0.04 (−0.80, 0.73)	0.888	−0.11 (−0.61, 0.40)	0.479
LDL-c (mg/dL)	−0.82 (−15.46, 13.82)	0.865	0.90 (−1.20, 3.00)	0.389	8.33 (−2.23, 18.90)	0.137	1.30 (−2.82, 5.41)	0.769	−22.81 (−50.34, 4.72)	0.130	2.30 (0.27, 4.31)	**0.020**	−1.60 (−2.91, −0.25)	**0.020**
Cholesterol (mg/dL)	−12.60 (−30.40, 5.20)	0.200	0.70 (−1.90, 3.26)	0.647	−5.33 (−18.31, 7.65)	0.373	4.31 (−0.70, 9.30)	0.103	4.96 (−29.00, 39.00)	0.768	−0.09 (−2.60, 2.42)	0.922	0.64 (−1.01, 2.30)	0.494
TG (mg/dL)	4.40 (−23.94, 32.70)	0.709	−3.77 (−7.80, 0.25)	0.081	−5.50 (−26.10, 15.10)	0.710	3.20 (−4.80, 11.11)	0.265	−29.73 (−83.24, 23.77)	0.291	1.70 (−2.27, 5.65)	0.434	−1.94 (−16.3, 19.91)	0.753
hsCRP (mg/L)	−0.31 (−0.84, 0.22)	0.214	−0.04 (−0.12, 0.03)	0.206	−0.10 (−0.50, 0.30)	0.521	0.14 (−0.01, 0.30)	0.252	0.34 (−0.66, 1.34)	0.421	0.02 (−0.05, 0.10)	0.457	0.01 (−0.04, 0.06)	0.974
IL-6 (pg/mL)	−13.75 (−80.05, 52.55)	0.758	−7.62 (−17.07, 1.83)	0.222	−32.26 (−80.22, 15.70)	0.265	−18.43 (−36.90, 0.003)	0.051	−65.00 (−190.3, 60.37)	0.510	−5.00 (−14.14, 4.40)	0.416	−7.92 (−14.00, −2.00)	**0.010**
IL-1*β* (pg/mL)	−0.30 (−1.00, 0.40)	0.373	−0.05 (−0.14, 0.04)	0.462	−0.37 (−0.84, 0.10)	0.154	−0.10 (−0.27, 0.09)	0.592	0.15 (−1.10, 1.40)	0.826	0.03 (−0.06, 0.12)	0.481	−0.02 (−0.10, 0.04)	0.720
TNF-α (pg/mL)	−12.12 (−24.71, −0.47)	**0.049**	−1.67 (−3.50, 0.14)	0.090	6.36 (−2.84, 15.55)	0.193	0.40 (−3.20, 4.00)	0.862	−4.52 (−28.63, 19.60)	0.673	1.22 (−0.56, 3.00)	0.152	−0.03 (−1.20, 1.14)	0.942
IL-10 (pg/mL)	−0.46 (−1.33, 0.42)	0.269	0.10 (−0.05, 0.20)	0.421	−0.30 (−0.93, 0.33)	0.197	0.23 (−0.02, 0.50)	0.232	1.02 (−0.63, 2.67)	0.297	−0.04 (−0.16, 0.08)	0.463	0.04 (−0.04, 0.12)	0.749
IL-4 (pg/mL)	−1.45 (−6.24, 3.34)	0.686	0.04 (−0.65, 0.73)	0.595	0.45 (−3.03, 3.94)	0.999	−0.96 (−2.30, 0.38)	0.371	8.61 (0.40, 17.60)	**0.032**	−0.10 (−0.77, 0.57)	0.888	0.12 (−0.32, 0.56)	0.966
TAC (μM)	0.64 (0.001, 1.27)	**0.044**	0.01 (−0.08, 0.10)	0.753	−0.22 (−0.70, 0.25)	0.395	0.06 (−0.12, 0.24)	0.536	−0.34 (−1.55, 0.88)	0.635	−0.07 (−0.16, 0.02)	0.125	−0.03 (−0.10, 0.03)	0.457
MDA (μM)	−0.51 (−1.50, 0.45)	0.318	−0.02 (−0.16, 0.12)	0.605	0.41 (−0.30, 1.11)	0.260	0.15 (−0.12, 0.43)	0.284	−0.70 (−2.51, 1.15)	0.444	0.08 (−0.06, 0.22)	0.297	0.05 (−0.04, 0.14)	0.403

^§^Adjusted for age, sex, smoking (yes/no), education (lower than high school diploma/higher than high school diploma), marital status (married/single/others), having regular physical activity (yes/no), immediate family members’ cancer history (yes/no), *H.pylori* (+/−), regular Aspirin or NSAIDs intake (yes/no), BMI, and total energy intake (kcal/day). FBG: fasting blood sugar; HDL-c: high-density lipoprotein cholesterol; LDL-c: low-density lipoprotein cholesterol; TG: triglycerides; hsCRP: high-sensitivity C-reactive protein; IL: interleukin; TNF-α: tumor necrosis factor alpha; TAC: total antioxidant capacity; MDA: malondialdehyde.

* All *p*-values were adjusted using the Benjamini–Hochberg correction method. *p*-values reaching statistical significance are shown in bold.

** All variables entered into the models as continuous variables.

In the fully adjusted linear regression models, there was an inverse and significant association between higher-than-median intakes of total carotenoids and LDL-c (*β*: −11.50, *p* = 0.015) and IL-6 (*β*: −34.1, *p* = 0.011), and between higher-than-median intakes of vitamin A and TNF-*α* (*β*: −8.2, *p* = 0.040; Table 4). On the other hand, there was a positive and significant association between higher-than-median intakes of lycopene and LDL-c (*β*: 10.9, *p* = 0.019; Table 4).

**Table 4:** Association (*β*-coefficients, 95% CI) between carotenoid and vitamin A intakes (as binary variables) and metabolic, inflammatory, and oxidative stress biomarkers, as determined by linear regression models^§^.

Variables***	Vitamin A (mg RAE/day) > 0.58***	*p*-values*	*β*-carotene (mg/d) > 4.66***	*p*-values*	α-carotene (mg/d) > 0.76***	*p*-values*	Lutein (mg/d) > 1.99***	*p*-values*	*β*-cryptoxanthin (mg/d) > 0.29***	*p*-values*	Lycopene (mg/d) > 4.89***	*p*-values*	Total carotenoids (mg/d) > 14.1***	*p*-values*
FBG (mg/dL)	0.10 (−7.30, 9.23)	0.814	0.86 (−7.53, 9.24)	0.841	1.27 (−6.99, 9.54)	0.761	1.30 (−6.97, 9.58)	0.756	0.73 (−7.48, 8.94)	0.860	−0.71 (−9.01, 7.61)	0.888	−1.06 (−9.50, 7.39)	0.806
HDL-c (mg/dL)	−1.39 (−4.83, 2.05)	0.426	2.57 (−0.91, 6.05)	0.146	1.98 (−1.46, 5.42)	0.257	−0.64 (−4.09, 2.82)	0.716	1.89 (−1.53, 5.30)	0.277	0.44 (−3.03, 3.91)	0.803	2.50 (−1.11, 6.00)	0.163
LDL-c (mg/dL)	3.16 (−6.03, 12.34)	0.498	−0.18 (−9.53, 9.18)	0.971	−4.31 (−13.51, 4.89)	0.356	−7.40 (−16.57, 1.76)	0.114	4.75 (−4.38, 13.9)	0.305	11.0 (1.84, 20.1)	**0.019**	−11.5 (−20.7, −2.21)	**0.015**
Cholesterol (mg/dL)	0.20 (−11.0, 11.4)	0.972	2.68 (−8.68, 14.1)	0.642	2.77 (−8.43, 14.0)	0.626	−5.58 (−16.8, 5.61)	0.326	−1.03 (−12.2, 10.1)	0.855	0.41 (−10.9, 11.7)	0.943	−8.22 (−19.6, 3.17)	0.156
TG (mg/dL)	−14.3 (−31.9, 3.41)	0.114	6.08 (−12.0, 24.2)	0.507	8.14 (−9.65, 26.0)	0.368	−2.56 (−20.4, 15.3)	0.777	7.03 (−10.7, 24.7)	0.434	−3.52 (−21.4, 14.4)	0.699	−0.34 (−18.6, 17.9)	0.970
hsCRP (mg/L)	0.10 (−0.24, 0.43)	0.569	0.01 (−0.33, 0.35)	0.951	0.18 (−0.15, 0.52)	0.273	−0.19 (−0.52, 0.15)	0.270	0.06 (−0.28, 0.39)	0.727	−0.23 (−0.57, 0.11)	0.176	0.07 (−0.27, 0.41)	0.688
IL-6 (pg/mL)	−13.2 (−54.8, 28.4)	0.523	−29.3 (−71.4, 12.8)	0.172	27.9 (−13.7, 69.3)	0.187	18.0 (−23.7, 59.7)	0.395	9.95 (−31.5, 51.4)	0.636	13.6 (−28.3, 55.5)	0.523	−34.2 (−76.5, −8.2)	**0.011**
IL-1*β* (pg/mL)	−0.07 (−0.47, 0.34)	0.750	0.23 (−0.18, 0.64)	0.267	0.21 (−0.20, 0.61)	0.310	−0.10 (−0.51, 0.30)	0.619	0.19 (−0.21, 0.59)	0.354	−0.22 (−0.62, 0.19)	0.291	0.22 (−0.20, 0.63)	0.304
TNF-α (pg/mL)	−8.19 (−16.0, −0.37)	**0.040**	−0.08 (−8.15, 8.00)	0.984	−3.84 (−11.8, 4.10)	0.340	−6.66 (−14.55, 1.23)	0.097	6.89 (−0.93, 14.71)	0.084	3.90 (−4.07, 11.86)	0.336	−1.23 (−6.89, 9.34)	0.766
IL-10 (pg/mL)	0.07 (−0.47, 0.61)	0.795	−0.06 (−0.61, 0.49)	0.828	0.19 (−0.35, 0.73)	0.487	−0.28 (−0.82, 0.27)	0.316	0.07 (−0.47, 0.61)	0.788	0.08 (−0.47, 0.63)	0.777	0.17 (−0.38, 0.73)	0.535
IL-4 (pg/mL)	−0.20 (−3.26, 2.86)	0.897	−1.41 (−4.51, 1.70)	0.373	0.11 (−3.00, 3.18)	0.946	2.02 (−1.04, 5.08)	0.193	−2.21 (−5.24, 0.82)	0.152	0.26 (−2.83, 3.35)	0.867	1.95 (−1.17, 5.08)	0.219
TAC (μM)	0.32 (−0.08, 0.71)	0.118	0.26 (−0.14, 0.67)	0.204	0.23 (−0.18, 0.63)	0.269	−0.03 (−0.44, 0.37)	0.870	0.12 (−0.28, 0.52)	0.566	0.23 (−0.17, 0.36)	0.253	0.20 (−0.21, 0.61)	0.327
MDA (μM)	0.05 (−0.55, 0.65)	0.869	−0.11 (−0.72, 0.50)	0.722	−0.09 (−0.69, 0.52)	0.777	−0.32 (−0.92, 0.28)	0.295	0.25 (−0.35, 0.84)	0.419	−0.29 (−0.90, 0.31)	0.344	−0.03 (−0.64, 0.59)	0.934

^§^ adjusted for age, sex, smoking (yes/no), education (lower than high school diploma/higher than higher school diploma), marital status (married/single/others), having regular physical activity (yes/no), immediate family members’ cancer history (yes/no), *H.pylori* (+/−), regular Aspirin or NSAIDs intake (yes/no), BMI, and total energy intake (kcal/day). FBG: fasting blood sugar; HDL-c: high-density lipoprotein cholesterol; LDL-c: low-density lipoprotein cholesterol; TG: triglycerides; hsCRP: high-sensitivity C-reactive protein; IL: interleukin; TNF-α: tumor necrosis factor alpha; TAC: total antioxidant capacity; MDA: malondialdehyde.

* All *p*-values were adjusted using the Benjamini–Hochberg correction method. *p*-values reaching statistical significance are shown in bold.

** Carotenoids and vitamin A intakes were entered into the models as binary variables.

*** Median (sample-specific cut-offs); values lower than the median are considered the reference group.

## 5 Discussion

In this case–control study, dietary intakes of total carotenoids, *β*-carotene, and vitamin A were associated with the odds of GC in crude, age- and sex-adjusted, and fully adjusted models, controlling for demographic, lifestyle, and clinical variables, as well as *H. pylori* infection, BMI, and total energy intake. Specifically, for total carotenoid intake (mg/d), *β*-carotene (mg/d), and vitamin A (mg RAE/d), when analyzed as continuous variables, an increase of one unit/day, corresponding, for example, to higher habitual consumption of carotenoid-rich foods such as carrots, spinach, or mixed vegetables, would translate into approximately 14, 23, and 71% lower odds of GC, respectively. A notable exception was lycopene, which was related not to a reduced but rather (non-significant) increase in the odds of GC. To our knowledge, this study is among the first to simultaneously evaluate dietary intakes of both total and individual carotenoids, along with vitamin A, in relation to GC, while integrating a comprehensive panel of circulating inflammatory and oxidative stress biomarkers within a single analytical framework.

Consistent with these estimates, carotenoid and vitamin A intakes were associated with biologically relevant differences in inflammatory, oxidative stress, and lipid biomarkers in fully adjusted linear regression models. For example, a 1 mg/day higher intake of lycopene and total carotenoids was associated with approximately 2.3 mg/dL higher and 1.6 mg/dL lower LDL-c levels, respectively. In comparison, total carotenoid intake was also associated with about 7.9 pg/mL lower IL-6 concentrations per 1 mg/day increase. Higher vitamin A intake was associated with lower TNF-α levels (−12.12 pg/mL per 1 mg RAE/day) and higher total antioxidant capacity (TAC; 0.64 μM per 1 mg RAE/day).

Surprisingly, in fully adjusted linear regression models, higher lycopene intake was positively associated with LDL-c concentrations. Consistently, participants with lycopene intake above the median (4.9 mg/day) exhibited almost 11 mg/dL higher LDL-c concentrations vs. those below. The positive association between lycopene intake and LDL-c warrants careful interpretation. Dietary sources of lycopene often include processed tomato products such as ketchup and pasta sauces, which may reflect rather processed foods, i.e., within a fast-food culture (13), along with increased intake of lipids, refined carbohydrates, and sodium (11, 13). In some earlier studies, we observed partial associations between higher lycopene intake and increased odds of obesity (11) and metabolic syndrome (13).

The observed inverse association between carotenoid and vitamin A intakes and the odds of GC in the fully adjusted models is broadly consistent with prior epidemiological evidence suggesting an inverse association between carotenoid-rich diets and gastric carcinogenesis (34–39). Several case–control, prospective cohort studies, and meta-analyses have reported lower GC risk with higher intakes of total carotenoids or carotenoid-rich foods. However, effect sizes and consistency vary across populations and exposure definitions (28, 34–41). Compared with studies focusing only on total carotenoids or specific ones such as *β*-carotene, the present findings highlight both individual and total carotenoids as relevant exposure metrics, aligning with observed inflammation/oxidative stress markers. While total carotenoids may better capture potentially synergistic effects of various carotenoids, reflecting likely a more varied dietary intake, individual carotenoids may be associated with contrasting dietary habits, such as lycopene, which is associated with a rather Westernized dietary pattern (11, 42–44).

However, the association of higher total and certain individual carotenoid intakes being paralleled by lower levels of systemic inflammation, including especially increased IL-6, TNF-*α*, and lower IL-4 in fully adjusted models, provides a biological context for the epidemiological findings. Indeed, similar findings have been shown in previous studies with cancer patients (24, 45–48), likely due to either direct antioxidant effects translating into lower inflammation, or via impacting cellular transcription factors such as Nrf-2 and NF-κB and thus downstream cytokine expression, as also shown in cancer patients (49–51). Also, IL-6 and TNF-α have been identified earlier as central mediators of chronic gastric inflammation and *H. pylori*–induced mucosal injury (24, 52, 53). In this regard, mixed carotenoid exposure has been shown to more effectively scavenge reactive oxygen species and limit oxidative DNA damage than single compounds alone (26, 27, 54).

Higher total carotenoid intake was also associated with lower LDL-c, likely reflecting a more plant-based diet rich in dietary fiber, which may reduce LDL-c (55); to our knowledge, there is no evidence that carotenoid supplements have been directly shown to reduce LDL-c. Such inverse associations between carotenoid intake and LDL-c have been reported earlier (55, 56) and have also been linked to gastric epithelial damage (37, 56–58). A notable exception in the present study was lycopene intake, which was again generally associated with higher LDL-c, suggesting that residual confounding from the diet also played a role in this observed association.

Interestingly, higher vitamin A intake was also associated with lower TNF-α and higher TAC. We can only speculate on the reasons, as vitamin A per se is not considered an antioxidant *in vivo* (59). Sufficient preformed vitamin A intake (such as from meat and fish) is important for optimal immune function (60), including mucosal immunity and epithelial integrity, with retinoids having been shown to suppress proinflammatory cytokine production (38, 61, 62). Earlier studies have already found associations with lower vitamin A intake and cancer, including GC (38).

Individual carotenoids may contribute to overall health and that of the digestive tract through partially overlapping but distinct biological pathways (50, 54). *β*-Carotene and *β*-cryptoxanthin, as provitamin A carotenoids, can be partially converted to retinoids that regulate cell differentiation, apoptosis, and immune responses via retinoic acid receptors (RXR), processes that are frequently dysregulated in gastric carcinogenesis (35, 63, 64). Lutein and *β*-cryptoxanthin are more polar xanthophylls, and their higher electrophilicity and solubility make them better candidates to interact with Nrf-2/Keap and NF-kB (16), suggesting that different carotenoids may act on complementary inflammatory/redox pathways relevant to GC development (65, 66).

The observed association between higher *H. pylori* infection and lower carotenoid levels in the present study is also noteworthy. Alterations in vitamin A-dependent signaling have been implicated in impaired gastric epithelial repair and increased susceptibility to chronic inflammation-driven carcinogenesis, particularly in the context of persistent *H. pylori* infection (67, 68). Similarly, as reviewed previously (19), astaxanthin at high doses in mice reduced gastric bacterial numbers of *H. pylori* and reduced mucosal inflammation, related to T-lymphocyte responses (69); though, *β*-carotene supplementation in patients up to 120 mg/d for 2 weeks was not found to be effective against *H. pylori* (70). However, reverse causality, namely that persons with *H. pylori* infection may consume less fruits due to bloating and other digestive problems, can also not be completely excluded.

Beyond the potential effects of carotenoids per se, it cannot be excluded that a large part of the observed associations are due to dietary confounders, as carotenoids coexist in the food matrix with other bioactive compounds, such as dietary fibers and phytochemicals that may act synergistically to modulate oxidative stress and inflammation (11, 71).

This study offers several strengths that enhance its scientific rigor and relevance. It employed a validated 168-item FFQ to comprehensively assess dietary intake of total and individual carotenoids as well as vitamin A, reducing measurement error. By integrating dietary data with a wide panel of metabolic, inflammatory, and oxidative stress biomarkers, the study provides mechanistic insights into potential pathways linking carotenoid intake to GC risk. Rigorous multivariable adjustment for major confounders such as age, sex, smoking, BMI, *H. pylori* infection, and total energy intake strengthens causal interpretation. Furthermore, analyzing both total and individual carotenoids allows for the evaluation of combined and specific effects, which were rarely addressed in previous research. Conducted in a high-risk region for GC, the study adds context-specific evidence that may inform prevention strategies.

Regarding limitations, a case–control design is inherently prone to recall bias, as dietary intake was self-reported and cases may have modified their diet before diagnosis. Second, the use of an FFQ, although validated, may lead to measurement error and misclassification of carotenoid and vitamin A intake. In addition, we acknowledge that differential recall between cases and controls cannot be completely excluded, and differential misclassification could have influenced the observed associations in either direction; the use of a validated FFQ does not eliminate this possibility. Third, the observational nature of the study precludes causal inference, and residual confounding from unmeasured factors, such as genetic predisposition, other dietary components, environmental factors, or socioeconomic variables, cannot be ruled out. Fourth, biomarker measurements were based on single-time-point blood samples, which may not fully capture long-term inflammatory or oxidative stress status. We also relied on carotenoid intake data rather than plasma levels. Carotenoid bioavailability depends on dietary factors such as fat co-ingestion, food processing (e.g., chopping, cooking), and the physical matrix (raw vs. processed), as well as host factors such as gastric acidity, mucosal integrity, and *H. pylori*-related inflammation (72–74). Thus, dietary data may not directly translate into bioavailable carotenoids. However, because plasma carotenoid concentrations primarily reflect dietary intake over the preceding few days, and the objective was to evaluate habitual long-term dietary patterns, the use of this dietary assessment by FFQ appears reasonable. Additionally, the study population was drawn from a specific region in Iran, which may limit generalizability to other populations with different dietary patterns and GC risk profiles. Finally, the modest sample size may have reduced statistical power to detect associations for some individual carotenoids. Thus, our findings should be interpreted with caution and confirmed in larger prospective cohorts incorporating repeated dietary assessments and serial measurements of plasma carotenoids, inflammatory biomarkers, and oxidative stress markers, which would help establish temporality, reduce measurement error, and better characterize long-term exposure and strengthen causal inference.

## 6 Conclusion

The present study emphasizes the biological plausibility that higher intakes of total carotenoids, certain individual carotenoids, and vitamin A, may be linked to lower GC odds through coordinated association with inflammation, oxidative stress, lipid metabolism, and immune regulation, key drivers of GC (50, 75). These findings support an inverse association between carotenoid-rich dietary patterns and gastric carcinogenesis, but cannot establish causality and should be interpreted in the context of potential recall bias, limitations of the FFQ, and residual confounding. Nevertheless, it was noteworthy that while lycopene intake showed a positive association with LDL-c, the overall pattern supports a beneficial effect of mixed carotenoid exposure rather than isolated compounds. The observed associations persisted after adjustment for major confounders, including *H. pylori* infection, lifestyle factors, and total energy intake, thereby strengthening the validity of these findings. From a public health perspective, promoting diets rich in carotenoid-containing foods, such as fruits and vegetables, may be an accessible and cost-effective strategy for GC prevention, particularly in high-risk populations. Further prospective studies and randomized controlled trials are warranted to confirm these associations and clarify dose–response relationships. Future research should also explore the interplay between carotenoid intake, genetic susceptibility, and microbiome composition to refine dietary recommendations for GC prevention.

## Data Availability

The datasets presented in this study can be found in online repositories. The names of the repository/repositories and accession number(s) can be found in the article/Supplementary material.
