# Supplementary material for: Dietary carotenoids and vitamin A are associated with lower gastric cancer risk and favorable inflammatory and oxidative stress biomarkers: an incident case– control study
Source: Front Nutr. 2026 Sep 7;13:1953378. doi: 10.3389/fnut.2026.1953378 (PMC13590419; doi:10.3389/fnut.2026.1953378)
Supplement: Supplementary file 1 [file Table_1.DOCX]

Supplementary Material

| **Supplementary Table 1**. Association between carotenoids and vitamin A intakes and odds (95%CI) of gastric cancer based on logistic regression models. | | | | | | |
| --- | --- | --- | --- | --- | --- | --- |
|  | Model A | p-values* | Model B | p-values* | Model C | p-values* |
| Vitamin A (mg RAE/d) (continuous variable) | 0.28 (0.09-0.84) | **0.023** | 0.31 (0.10-0.94) | **0.039** | 0.29 (0.09-0.95) | **0.040** |
| Vitamin A (mg RAE/d) ≤ 0.58** | Ref | 0.740 | Ref | 0.408 | Ref | 0.414 |
| Vitamin A (mg RAE/d) > 0.58** | 0.74 (0.41-1.34) |  | 0.78 (0.43-1.41) |  | 0.76 (0.40-1.46) |  |
| β-Carotene (mg/d) (continuous variable) | 0.77 (0.66-0.89) | **<0.001** | 0.75 (0.65-0.88) | **<0.001** | 0.77 (0.65-0.90) | **<0.001** |
| β-Carotene (mg/d) ≤ 4.66** | Ref | **0.009** | Ref | **0.009** | Ref | **0.010** |
| β-Carotene (mg/d) > 4.66** | 0.45 (0.24-0.82) |  | 0.44 (0.24-0.81) |  | 0.41 (0.21-0.81) |  |
| α-Carotene (mg/d) (continuous variable) | 0.77 (0.38-1.55) | 0.470 | 0.77 (0.38-1.571) | 0.475 | 0.93 (0.42-2.03) | 0.848 |
| α-Carotene (mg/d) ≤ 0.76** | Ref | 0.709 | Ref | 0.202 | Ref | 0.654 |
| α-Carotene (mg/d) > 0.76** | 0.71 (0.39-1.28) |  | 0.67 (0.37-1.23) |  | 0.86 (0.45-1.65) |  |
| Lutein (mg/d) (continuous variable) | 1.00 (0.77-1.27) | 0.929 | 0.97 (0.75-1.25) | 0.804 | 0.96 (0.72-1.27) | 0.780 |
| Lutein (mg/d) ≤ 1.99** | Ref | 0.330 | Ref | 0.247 | Ref | 0.062 |
| Lutein (mg/d) > 1.99** | 0.74 (0.41-1.35) |  | 0.70 (0.38-1.28) |  | 0.53 (0.27-1.03) |  |
| β-Cryptoxanthin (mg/d) (continuous variable) | 0.63 (0.10-3.89) | 0.620 | 0.71 (0.11-4.47) | 0.712 | 0.81 (0.12-5.66) | 0.828 |
| β-Cryptoxanthin (mg/d) ≤ 0.29** | Ref | 0.257 | Ref | 0.318 | Ref | 0.625 |
| β-Cryptoxanthin (mg/d) > 0.29** | 0.71 (0.39-1.28) |  | 0.74 (0.41-1.34) |  | 0.85 (0.449-1.62) |  |
| Lycopene (mg/d) (continuous variable) | 1.02 (0.89-1.16) | 0.777 | 1.04 (0.91-1.18) | 0.591 | 1.05 (0.91-1.21) | 0.479 |
| Lycopene (mg/d) ≤ 4.89** | Ref | 0.710 | Ref | 0.649 | Ref | 0.460 |
| Lycopene (mg/d) > 4.89** | 1.118 (0.619-2.020) |  | 1.150 (0.630-2.097) |  | 1.280 (0.666-2.460) |  |
| Total carotenoids (mg/d) (continuous variable) | 0.91 (0.83-0.99) | **0.033** | 0.91 (0.83-0.99) | **0.046** | 0.86 (0.81-0.98) | **0.034** |
| Total carotenoids (mg/d) ≤ 14.1** | Ref | 0.083 | Ref | 0.124 | Ref | 0.370 |
| Total carotenoids (mg/d) > 14.1** | 0.59 (0.32-1.10) |  | 0.62 (0.34-1.140) |  | 0.73 (0.37-1.45) |  |
| Model A: crude model, without any further adjustment,  Model B: adjusted for age and sex,  Model C: adjusted for age, sex, smoking (yes/no), education (lower than high school diploma/higher than higher school diploma), marital status (married/single/others), having regular physical activity (yes/no), immediate family members' cancer history (yes/no), *H. pylori* (+/-), regular Aspirin or NSAIDs intake (yes/no), BMI, and total energy intake (kcal/day).  * All p-values were adjusted using the Benjamini-Hochberg correction method.  BMI: body mass index, Ref: reference group.  ** median | | | | | | |

| **Supplementary Table 2**. Association (β-coefficients, 95%CI) between carotenoid and vitamin A intakes and metabolic, inflammatory, and oxidative stress biomarkers, as determined by linear regression models^§^. | | | | | | | | | | | | | | |
| --- | --- | --- | --- | --- | --- | --- | --- | --- | --- | --- | --- | --- | --- | --- |
| Variables** | Vitamin A (mg RAE/day) | p-values* | β-carotene (mg/d) | p-values* | α-carotene (mg/d) | p-values* | Lutein (mg/d) | p-values* | β-cryptoxanthin (mg/d) | p-values* | Lycopene (mg/d) | p-values* | Total carotenoids (mg/d) | p-values* |
| FBG (mg/dL) | -0.42 (-13.20, 12.30) | 0.944 | -0.21 (-2.04, 1.63) | 0.826 | 0.26 (-9.04, 9.56) | 0.956 | -1.45 (-4.85, 1.95) | 0.400 | -1.00 (-25.36, 23.35) | 0.935 | 0.77 (-0.99, 2.53) | 0.388 | 0.09 (-1.07, 1.24) | 0.888 |
| HDL-c (mg/dL) | 0.38 (-4.93, 5.70) | 0.888 | -0.43 (-1.19, 0.33) | 0.268 | -0.92 (-4.80, 2.96) | 0.642 | 0.97 (-0.45, 2.38) | 0.179 | -10.40 (-20.44, -0.35) | **0.043** | 0.07 (-0.66, 0.81) | 0.850 | 1.40 (0.05, 2.61) | **0.050** |
| LDL-c (mg/dL) | -6.10 (-20.61, 8.42) | 0.408 | 0.94 (-1.15, 3.03) | 0.375 | 7.97 (-2.60, 18.52) | 0.138 | -0.10 (-4.00, 3.80) | 0.959 | -24.48 (-52.06, 3.10) | 0.082 | 2.02 (0.03, 4.01) | **0.047** | -0.07 (-0.55, 0.42) | 0.786 |
| Cholesterol (mg/dL) | -10.86 (-28.19, 6.46) | 0.217 | 0.71 (-1.80, 3.21) | 0.577 | -4.50 (-17.19, 8.18) | 0.484 | 5.06 (0.47, 9.65) | **0.031** | 8.92 (-24.32, 42.17) | 0.597 | 0.46 (-1.94, 2.87) | 0.703 | 1.02 (-0.55, 2.59) | 0.204 |
| TG (mg/dL) | 4.76 (-22.71, 32.24) | 0.733 | -3.65 (-7.65, 0.27) | 0.068 | -6.38 (-26.43, 13.66) | 0.530 | 4.73 (-2.58, 12.04) | 0.204 | -25.36 (-77.78, 27.05) | 0.341 | 1.47 (-2.32, 5.27) | 0.444 | -0.43 (-2.92, 2.07) | 0.737 |
| hsCRP (mg/L) | -0.32 (-0.84, 0.20) | 0.227 | -0.05 (-0.12, 0.03) | 0.191 | -0.20 (-0.57, 0.19) | 0.317 | 0.10 (-0.04, 0.24) | 0.148 | 0.43 (-0.56, 1.42) | 0.393 | 0.03 (-0.05, 0.10) | 0.501 | -0.001 (-0.05, 0.05) | 0.979 |
| IL-6 (pg/mL) | -22.41 (-89.83, 45.1) | 0.514 | -8.46 (-18.10, 1.17) | 0.085 | -36.81 (-85.80, 12.17) | 0.140 | -16.76 (-34.64, 1.11) | 0.066 | -37.74 (-166.7, 91.21) | 0.564 | -5.43 (-14.72, 3.87) | 0.251 | -8.30 (-14.30, -2.30) | **0.007** |
| IL-1β (pg/mL) | -0.30 (-0.92, 0.35) | 0.375 | -0.04 (-0.14, 0.05) | 0.383 | -0.36 (-0.83, 0.10) | 0.121 | -0.07 (-0.24, 0.10) | 0.400 | 0.10 (-1.11, 1.32) | 0.866 | -0.01 (-0.08, 0.10) | 0.866 | -0.03 (-0.08, 0.03) | 0.363 |
| TNF-α (pg/mL) | -10.60 (-22.74, 1.54) | 0.086 | -1.75 (-3.50, -0.01) | **0.049** | 5.22 (-3.70, 14.12) | 0.248 | 0.14 (-3.12, 3.42) | 0.931 | -3.52 (-26.91, 19.87) | 0.767 | 1.07 (-0.61, 2.75) | 0.211 | -0.15 (-1.25, 0.96) | 0.797 |
| IL-10 (pg/mL) | -0.40 (-1.25, 0.50) | 0.381 | 0.09 (-0.04, 0.21) | 0.178 | -0.31 (-0.94, 0.32) | 0.336 | 0.21 (-0.02, 0.44) | 0.076 | 1.11 (-0.54, 2.76) | 0.184 | -0.02 (-0.14, 0.10) | 0.772 | 0.05 (-0.03, 0.12) | 0.228 |
| IL-4 (pg/mL) | -1.50 (-6.24, 3.30) | 0.543 | 0.05 (-0.63, 0.74) | 0.876 | -0.02 (-3.50, 3.47) | 0.992 | -0.84 (-2.11, 0.43) | 0.191 | 9.25 (0.23, 18.27) | **0.045** | -0.08 (-0.74, 0.57) | 0.802 | -0.09 (-0.52, 0.34) | 0.678 |
| TAC (μM) | 0.55 (-0.07, 1.17) | 0.083 | 0.02 (-0.07 0.11) | 0.681 | -0.21 (-0.67, 0.24) | 0.361 | 0.06 (-0.11, 0.23) | 0.495 | -0.35 (-1.55, 0.85) | 0.566 | -0.06 (-0.14, 0.03) | 0.183 | -0.01 (-0.07, 0.04) | 0.602 |
| MDA (μM) | -0.51 (-1.44, 0.42) | 0.279 | -0.01 (-0.15, 0.12) | 0.849 | 0.32 (-0.36, 1.00) | 0.350 | 0.17 (-0.08, 0.42) | 0.186 | -0.71 (-2.50, 1.80) | 0.435 | 0.05 (-0.07, 0.18) | 0.411 | 0.04 (-0.04, 0.12) | 0.344 |
| § crude model, without any further adjustment.  ** All variables entered into the models as continuous variables.  * All p-values were adjusted using the Benjamini-Hochberg correction method.  BMI: body mass index; FBG: fasting blood sugar; HDL-c: high-density lipoprotein cholesterol; LDL-c: low-density lipoprotein cholesterol; TG: triglycerides; hsCRP: high-sensitivity C-reactive protein; IL: interleukin; TNF-α: tumor necrosis factor alpha; TAC: total antioxidant capacity; MDA: malondialdehyde. | | | | | | | | | | | | | | |

| **Supplementary Table 3**. Association (β-coefficients, 95% CI) between carotenoid and vitamin A intakes (as binary variables) and metabolic, inflammatory, and oxidative stress biomarkers, as determined by linear regression models^§^. | | | | | | | | | | | | | | |
| --- | --- | --- | --- | --- | --- | --- | --- | --- | --- | --- | --- | --- | --- | --- |
| Variables** | Vitamin A (mg RAE/day) > 0.58*** | p-values* | β-carotene (mg/d) > 4.66*** | p-values* | α-carotene (µg/d) > 0.76*** | p-values* | Lutein (µg/d) > 1.99*** | p-values* | β-cryptoxanthin (µg/d) > 0.29*** | p-values* | Lycopene (µg/d) > 4.89*** | p-values* | Total carotenoids (µg/d) > 14.1** | p-values* |
| FBG (mg/dL) | 0.16 (-7.79, 8.12) | 0.968 | 0.37 (-7.58, 8.33) | 0.927 | 1.64 (-6.32, 9.59) | 0.685 | 0.69 (-7.27, 8.65) | 0.865 | 0.37 (-7.59, 8.33) | 0.927 | -1.71 (-9.66, 6.25) | 0.672 | -1.84 (-9.79, 6.11) | 0.648 |
| HDL-c (mg/dL) | -1.71 (-5.02, 1.60) | 0.310 | 2.05 (-1.26, 5.35) | 0.222 | 1.88 (-1.42, 5.20) | 0.262 | -1.01 (-4.33, 2.31) | 0.555 | 1.93 (-1.38, 5.24) | 0.251 | 0.31 (-3.02, 363) | 0.857 | 1.64 (-1.68, 4.95) | 0.331 |
| LDL-c (mg/dL) | 5.61 (-3.44, 14.66) | 0.223 | 0.88 (-8.21, 9.97) | 0.848 | -5.01 (-14.07, 4.05) | 0.277 | -6.51 (-15.54, 2.53) | 0.157 | 4.57 (-4.49, 13.64) | 0.321 | 11.08 (2.14, 20.01) | **0.015** | -8.63 (-17.62, 0.37) | 0.060 |
| Cholesterol (mg/dL) | 0.11 (-10.8, 11.0) | 0.985 | 1.20 (-9.67, 12.1) | 0.827 | 2.54 (-8.33, 13.4) | 0.646 | -7.39 (-18.2, 3.43) | 0.180 | -1.03 (-11.9, 9.84) | 0.851 | -0.40 (-11.3, 10.5) | 0.942 | -10.6 (-21.4, 0.14) | 0.053 |
| TG (mg/dL) | -12.8 (-29.8, 4.28) | 0.141 | 6.25 (-10.9, 23.4) | 0.473 | 10.6 (-6.55, 27.65) | 0.225 | -4.55 (-21.7, 12.6) | 0.602 | 5.35 (-11.8, 22.5) | 0.539 | -3.89 (-21.1, 13.3) | 0.655 | 0.58 (-16.6, 17.8) | 0.067 |
| hsCRP (mg/L) | 0.09 (-0.23, 0.42) | 0.576 | -0.0001 (-0.33, 0.32) | 0.999 | 0.23 (-0.10, 0.55) | 0.169 | -0.22 (-0.54, 0.11) | 0.184 | 0.07 (-0.25, 0.40) | 0.652 | -0.22 (-0.54, 0.11) | 0.185 | 0.08 (-0.24, 0.41) | 0.625 |
| IL-6 (pg/mL) | -17.3 (-59.4, 24.8) | 0.419 | -44.9 (-86.5, -3.23) | **0.035** | 35.1 (-6.74, 77.0) | 0.100 | 24.2 (-17.8, 66.2) | 0.257 | 6.63 (-35.5, 48.8) | 0.757 | 12.0 (-30.1, 54.2) | 0.574 | -49.7 (-91.2, -8.1) | **0.019** |
| IL-1β (pg/mL) | -0.09 (-0.50, 0.31) | 0.659 | 0.29 (-0.11, 0.68) | 0.156 | 0.18 (-0.22, 0.58) | 0.374 | 0.02 (-0.38, 0.41) | 0.936 | 0.22 (-0.17, 0.62) | 0.270 | -0.13 (-0.53, 0.27) | 0.517 | 0.32 (-0.07, 0.72) | 0.111 |
| TNF-α (pg/mL) | -7.09 (-14.7, 0.48) | 0.066 | -1.10 (-8.74, 6.55) | 0.777 | -3.26 (-11.0, 4.37) | 0.400 | -5.41 (-13.0, 2.20) | 0.162 | 6.76 (-0.81, 14.4) | 0.080 | 2.77 (-4.86, 10.4) | 0.475 | -2.21 (-9.85, 5.43) | 0.569 |
| IL-10 (pg/mL) | 0.01 (-0.54, 0.55) | 0.980 | -0.20 (-0.74, 0.34) | 0.467 | 0.17 (-0.37, 0.71) | 0.529 | -0.38 (-0.92, 0.16) | 0.169 | 0.01 (-0.54, 0.55) | 0.983 | 0.08 (-0.46, 062) | 0.772 | -0.07 (-0.61, 0.47) | 0.802 |
| IL-4 (pg/mL) | 0.38 (-2.60, 3.36) | 0.800 | -0.68 (-3.66, 2.30) | 0.654 | 0.18 (-2.80, 3.16) | 0.904 | 2.08 (-0.88, 5.05) | 0.167 | -2.42 (-5.38, 0.54) | 0.108 | 0.05 (-2.94, 3.03) | 0.975 | 2.40 (-0.57, 5.36) | 0.111 |
| TAC (μM) | 0.25 (-0.14, 0.64) | 0.214 | 0.22 (-0.17, 0.61) | 0.268 | 0.19 (-0.21, 0.58) | 0.350 | -0.05 (-0.44, 0.34) | 0.800 | 0.14 (-0.26, 0.528) | 0.479 | 0.20 (-0.19, 0.59) | 0.321 | 0.18 (-0.22, 0.57) | 0.378 |
| MDA (μM) | 0.09 (-0.494, 0.675) | 0.761 | -0.26 (-0.84, 0.33) | 0.382 | -0.05 (-0.64, 0.53) | 0.863 | -0.39 (-0.97, 0.19) | 0.187 | 0.30 (-0.29, 0.90) | 0.318 | -0.21 (-0.791, 0378) | 0.486 | -0.07 (-0.65, 0.52) | 0.816 |
| § crude model, without any further adjustment.  **Carotenoids and vitamin A intakes were entered into the models as binary variables.  * All p-values were adjusted using the Benjamini-Hochberg correction method.  *** Median; values lower than the median were considered the reference group.  FBG: fasting blood sugar; HDL-c: high-density lipoprotein cholesterol; LDL-c: low-density lipoprotein cholesterol; TG: triglycerides; hsCRP: high-sensitivity C-reactive protein; IL: interleukin; TNF-α: tumor necrosis factor alpha; TAC: total antioxidant capacity; MDA: malondialdehyde. | | | | | | | | | | | | | | |

| **Supplementary Table 4**. Association (β-coefficients, 95%CI) between carotenoid and vitamin A intakes and metabolic, inflammatory, and oxidative stress biomarkers, as determined by linear regression models^§^. | | | | | | | | | | | | | | |
| --- | --- | --- | --- | --- | --- | --- | --- | --- | --- | --- | --- | --- | --- | --- |
| Variables** | Vitamin A (mg RAE/day) | p-values* | β-carotene (mg/d) | p-values* | α-carotene (mg/d) | p-values* | Lutein (mg/d) | p-values* | β-cryptoxanthin (mg/d) | p-values* | Lycopene (mg/d) | p-values* | Total carotenoids (mg/d) | p-values* |
| FBG (mg/dL) | -0.96 (-13.91, 11.98) | 0.888 | -0.12 (-1.97, 1.72) | 0.894 | 0.54 (-8.82, 9.88) | 0.910 | -1.37 (-4.82, 2.10) | 0.434 | -0.37 (-24.98, 24.24) | 0.977 | 0.74 (-1.04, 2.51) | 0.414 | 0.12 (-1.05, 1.29) | 0.842 |
| HDL-c (mg/dL) | -0.18 (-5.57, 5.21) | 0.948 | -0.42 (-1.20, 0.34) | 0.277 | -1.00 (-4.90, 2.89) | 0.612 | 0.87 (-0.56, 2.31) | 0.230 | -11.24 (-21.35, -1.14) | **0.029** | 0.003 (-0.74, 0.75) | 0.993 | 0.11 (-0.60, 0.38) | 0.657 |
| LDL-c (mg/dL) | -3.34 (-18.00, 11.21) | 0.651 | 0.90 (-1.17, 3.00) | 0.392 | 8.41 (-2.03, 18.86) | 0.114 | 0.48 (-3.41, 4.36) | 0.809 | -21.35 (-48.85, 6.14) | 0.127 | 2.41 (0.43, 4.38) | **0.017** | -1.54 (-2.84, -0.24) | **0.020** |
| Cholesterol (mg/dL) | -12.05 (-29.53, 5.44) | 0.176 | 0.58 (-1.92, 3.10) | 0.646 | -5.33 (-18.00, 7.35) | 0.408 | 4.57 (-0.07, 9.21) | **0.053** | 5.52 (-28.00, 39.00) | 0.745 | 0.34 (-2.10, 2.76) | 0.782 | 0.84 (-0.75, 2.43) | 0.298 |
| TG (mg/dL) | 3.93 (-24.05, 31.90) | 0.782 | -3.62 (-7.57, 0.34) | 0.073 | -6.36 (-26.56, 13.85) | 0.535 | 4.76 (-2.67, 12.20) | 0.208 | -26.20 (-79.27, 26.90) | 0.333 | 1.40 (-2.45, 5.25) | 0.475 | -0.47 (-3.00, 2.07) | 0.718 |
| hsCRP (mg/L) | -0.31 (-0.84, 0.22) | 0.245 | -0.05 (-0.12, 0.03) | 0.190 | -0.20 (-0.57, 0.20) | 0.324 | 0.11 (-0.03, 0.25) | 0.135 | 0.45 (-0.55, 1.46) | 0.374 | 0.03 (-0.05, 0.10) | 0.469 | 0.002 (-0.05, 0.05) | 0.945 |
| IL-6 (pg/mL) | -7.38 (-74.60, 59.83) | 0.829 | -8.96 (-18.46, 0.53) | 0.064 | -35.64 (-83.92, 12.64) | 0.147 | -14.53 (-32.33, 3.30) | 0.109 | -22.50 (-150.3, 105.3) | 0.729 | -3.68 (-12.93, 5.56) | 0.433 | -7.50 (-14.00, -1.51) | **0.014** |
| IL-1β (pg/mL) | -0.20 (-0.83, 0.45) | 0.557 | -0.04 (-0.14, 0.05) | 0.346 | -0.36 (-0.82, 0.10) | 0.127 | -0.06 (-0.23, 0.11) | 0.509 | 0.21 (-1.01, 1.43) | 0.736 | 0.02 (-0.07, 0.11) | 0.659 | -0.02 (-0.08, 0.04) | 0.479 |
| TNF-α (pg/mL) | -11.16 (-23.52, 1.20) | 0.076 | -1.76 (-3.52, -0.01) | **0.049** | 5.20 (-3.78, 14.16) | 0.255 | 0.08 (-3.25, 3.40) | 0.963 | -3.99 (-27.70, 19.71) | 0.740 | 1.06 (-0.65, 2.80) | 0.224 | -0.17 (-1.30, 0.96) | 0.762 |
| IL-10 (pg/mL) | -0.48 (-1.35, 0.38) | 0.271 | 0.08 (-0.04, 0.20) | 0.206 | -0.36 (-0.98, 0.26) | 0.258 | 0.17 (-0.06, 0.40) | 0.145 | 0.90 (-0.76, 2.53) | 0.290 | -0.03 (-0.15, 0.09) | 0.619 | 0.04 (-0.04, 0.11) | 0.380 |
| IL-4 (pg/mL) | -1.58 (-6.42, 3.27) | 0.522 | 0.08 (-0.61, 0.77) | 0.814 | 0.10 (-3.41, 3.60) | 0.956 | -0.80 (-2.10, 0.50) | 0.224 | 9.76 (0.66, 18.87) | **0.036** | -0.10 (-0.76, 0.60) | 0.795 | -0.07 (-0.51, 0.36) | 0.743 |
| TAC (μM) | 0.63 (0.004, 1.26) | **0.048** | 0.27 (-0.06, 0.12) | 0.549 | -0.20 (-0.64, 0.27) | 0.415 | 0.07 (-0.10, 0.24) | 0.427 | -0.31 (-1.51, 0.90) | 0.612 | -0.07 (-0.15, 0.02) | 0.133 | -0.01 (-0.07, 0.04) | 0.645 |
| MDA (μM) | -0.50 (-1.44, 0.45) | 0.303 | -0.02 (-0.15, 0.12) | 0.783 | 0.31 (-0.38, 0.99) | 0.382 | 0.16 (-0.10, 0.41) | 0.209 | -0.77 (-2.58, 1.03) | 0.399 | 0.06 (-0.07, 0.20) | 0.385 | 0.04 (-0.05, 0.12) | 0.372 |
| § adjusted for age and sex.  ** All variables entered into the models as continuous variables.  * All p-values were adjusted using the Benjamini-Hochberg correction method.  FBG: fasting blood sugar; HDL-c: high-density lipoprotein cholesterol; LDL-c: low-density lipoprotein cholesterol; TG: triglycerides; hsCRP: high-sensitivity C-reactive protein; IL: interleukin; TNF-α: tumor necrosis factor alpha; TAC: total antioxidant capacity; MDA: malondialdehyde. | | | | | | | | | | | | | | |

| **Supplementary Table 5**. Association (β-coefficients, 95% CI) between carotenoid and vitamin A intakes (as binary variables) and metabolic, inflammatory, and oxidative stress biomarkers, as determined by linear regression models^§^. | | | | | | | | | | | | | | |
| --- | --- | --- | --- | --- | --- | --- | --- | --- | --- | --- | --- | --- | --- | --- |
| Variables** | Vitamin A (mg RAE/day) > 0.58*** | p-values* | β-carotene (mg/d) > 4.66*** | p-values* | α-carotene (mg/d) > 0.76*** | p-values* | Lutein (mg/d) > 1.99*** | p-values* | β-cryptoxanthin (mg/d) > 0.29*** | p-values* | Lycopene (mg/d) > 4.89*** | p-values* | Total carotenoids (mg/d) > 14.1** | p-values* |
| FBG (mg/dL) | 0.46 (-7.56, 8.49) | 0.909 | -0.002 (-8.04, 8.04) | 0.999 | 1.49 (-6.51, 9.49) | 0.714 | 0.69 (-7.33, 8.72) | 0.865 | 0.51 (-7.50, 8.52) | 0.900 | -1.26 (-9.30, 6.77) | 0.757 | -2.17 (-10.30, 5.95) | 0.598 |
| HDL-c (mg/dL) | -1.53 (-4.86, 1.81) | 0.367 | 2.21 (-1.13, 5.54) | 0.193 | 1.75 (-1.58, 5.07) | 0.301 | -0.78 (-4.12, 2.56) | 0.645 | 2.13 (-1.19, 5.45) | 0.207 | 0.33 (-3.01, 3.68) | 0.845 | 2.09 (-1.28, 5.46) | 0.223 |
| LDL-c (mg/dL) | 4.65 (-4.35, 13.65) | 0.309 | 0.24 (-8.80, 9.28) | 0.958 | -4.26 (-13.24, 4.72) | 0.350 | -7.79 (-16.7, 1.16) | 0.088 | 3.66 (-5.33, 12.7) | 0.422 | 11.4 (2.49, 20.2) | **0.012** | -10.9 (-19.9, -1.94) | **0.017** |
| Cholesterol (mg/dL) | 0.18 (-10.7, 11.1) | 0.974 | 2.39 (-8.52, 13.3) | 0.666 | 2.35 (-8.51, 13.2) | 0.669 | -6.69 (-17.5, 4.17) | 0.226 | -0.71 (-11.6, 10.2) | 0.897 | -1.25 (-12.2, 9.66) | 0.822 | -9.24 (-20.2, 1.72) | 0.098 |
| TG (mg/dL) | -12.5 (-29.8, 4.74) | 0.154 | 6.29 (-11.1, 23.7) | 0.476 | 10.4 (-6.90, 27.6) | 0.238 | -4.32 (-21.7, 13.0) | 0.624 | 5.68 (-11.6, 23.0) | 0.518 | -3.65 (-21.0, 13.7) | 0.679 | 0.92 (-16.7, 18.5) | 0.918 |
| hsCRP (mg/L) | 0.09 (-0.24, 0.42) | 0.598 | -0.003 (-0.33, 0.33) | 0.984 | 0.23 (-0.10, 0.56) | 0.163 | -0.23 (-0.55, 0.10) | 0.172 | 0.07 (-0.26, 0.38) | 0.637 | -0.22 (-0.55, 0.11) | 0.182 | 0.07 (-0.26, 0.41) | 0.662 |
| IL-6 (pg/mL) | -11.7 (-53.4, 30.0) | 0.580 | -43.2 (-84.4, -1.90) | **0.040** | 39.5 (-1.67, 80.6) | 0.060 | 18.5 (-23.1, 60.1) | 0.381 | 1.70 (-39.9, 43.3) | 0.936 | 10.4 (-31.3, 52.2) | 0.622 | -42.4 (-84.1, -0.58) | **0.047** |
| IL-1β (pg/mL) | -0.13 (-0.53, 0.27) | 0.525 | 0.27 (-0.12, 0.67) | 0.174 | 0.21 (-0.19, 0.60) | 0.302 | -0.02 (-0.42, 0.38) | 0.910 | 0.19 (-0.21, 0.59) | 0.343 | -0.14 (-0.54, 0.25) | 0.475 | 0.27 (-0.13, 0.68) | 0.180 |
| TNF-α (pg/mL) | -7.30 (-14.9, 0.39) | 0.063 | -1.21 (-8.952, 6.534) | 0.758 | -3.356 (-11.048, 4.335) | 0.390 | -5.335 (-13.025, 2.355) | 0.173 | 6.907 (-0.738, 14.552) | 0.076 | 2.824 (-4.906, 10.554) | 0.427 | -2.527 (-5.299, 10.352) | 0.525 |
| IL-10 (pg/mL) | 0.03 (-0.51, 0.56) | 0.927 | -0.13 (-0.67, 0.41) | 0.637 | 0.15 (-0.39, 0.70) | 0.557 | -0.32 (-0.86, 0.22) | 0.239 | 0.04 (-0.50, 0.58) | 0.891 | 0.04 (-0.50, 0.58) | 0.895 | 0.06 (-0.49, 0.60) | 0.842 |
| IL-4 (pg/mL) | 0.45 (-2.55, 3.46) | 0.766 | -0.85 (-3.86, 2.16) | 0.578 | 0.16 (-2.84, 3.16) | 0.917 | 2.06 (-0.93, 5.05) | 0.176 | -2.43 (-5.40, 0.56) | 0.111 | 0.21 (-2.80, 3.22) | 0.890 | 2.31 (-0.72, 5.34) | 0.133 |
| TAC (μM) | 0.29 (-0.11, 0.68) | 0.150 | 0.19 (-0.20, 0.58) | 0.341 | 0.17 (-0.22, 0.56) | 0.398 | -0.05 (-0.44, 0.35) | 0.822 | 0.16 (-0.24, 0.55) | 0.435 | 0.25 (-0.14, 0.64) | 0.212 | 0.17 (-0.23, 0.56) | 0.407 |
| MDA (μM) | 0.07 (-0.52, 0.66) | 0.811 | -0.23 (-0.82, 0.36) | 0.435 | -0.04 (-0.63, 0.55) | 0.888 | -0.39 (-0.98, 0.20) | 0.189 | 0.29 (-0.30, 0.88) | 0.333 | -0.24 (-0.83, 0.35) | 0.416 | -0.05 (-0.65, 0.55) | 0.871 |
| § adjusted for age and sex.  **Carotenoids and vitamin A intakes were entered into the models as binary variables.  * All p-values were adjusted using the Benjamini-Hochberg correction method.  *** Median; values below the median were considered the reference group.  FBG: fasting blood sugar; HDL-c: high-density lipoprotein cholesterol; LDL-c: low-density lipoprotein cholesterol; TG: triglycerides; hsCRP: high-sensitivity C-reactive protein; IL: interleukin; TNF-α: tumor necrosis factor alpha; TAC: total antioxidant capacity; MDA: malondialdehyde. | | | | | | | | | | | | | | |
